# Correlations between Visual Performance and Chorioretinal Variables after Vitrectomy for the Idiopathic Macular Hole

**DOI:** 10.1155/2022/6641956

**Published:** 2022-12-28

**Authors:** Minfeng Chen, Chenchen Zheng, Yu Liu, Feng Chen, Dan Xu, Fan Lu

**Affiliations:** School of Ophthalmology and Optometry, Wenzhou Medical University, Wenzhou 325000, Zhejiang, China

## Abstract

**Purpose:**

To investigate the relationships between visual function and the retinal and choroid microstructure in idiopathic macular hole patients after surgery.

**Methods:**

A prospective study investigated changes in the fundus structure and visual function in 16 macular hole patients before and after surgery. Patients' best-corrected visual acuity (BCVA) and retinal sensitivity were measured by an EDTRS visual chart and microperimetry (MP1), respectively. The thickness of the retina and the blood supply to the retina and superficial choroid were detected by OCTA, and the choroidal capillary blood flow density was analysed with MATLAB. The thickness of the choroid and the aperture size of the macular hole were detected by Heidelberg OCT.

**Results:**

Compared with before surgery and one month after surgery, the BCVA (3 months: 0.47 ± 0.27, before: 1.02 ± 0.22, 1 month: 0.66 ± 0.27, and *P* < 0.05) and the central sensitivity of the retina (3 months: 14.88 ± 2.87 dB, before: 8.76 ± 3.27 dB, 1 month: 12.22 ± 3.30 dB, and *P* < 0.05) were significantly improved three months after surgery. The change in BCVA was significantly correlated with the basal diameter (*r* = 0.677 and *P* = 0.004), the minimum diameter (*r* = 0.585 and *P* = 0.017), the macular hole cystoid height area index (*r* = −0.618 and *P* = 0.011), the central macular hole index (*r* = −0.727 and *P* = 0.001), the peripheral macular hole index (*r* = −0.758 and *P* = 0.001), the central tractional hole index (*r* = −0.717 and *P* = 0.002), the peripheral tractional hole index (*r* = −0.725 and *P* = 0.001), and changes in the peripheral blood vessel density of the choroid capillary layer (*r* = 0.585 and *P* = 0.0017). The change in central retinal sensitivity was correlated with the change in the superficial foveal avascular zone (FAZ; *r* = 0.520 and *P* = 0.039), change in the retinal superficial peripheral blood flow density (*r* = −0.503 and *P* = 0.047), change in the deep FAZ (*r* = 0.599 and *P* = 0.014), and change in the retinal deep peripheral blood flow density (*r* = −0.601 and *P* = 0.014).

**Conclusions:**

The morphology of the macular hole as well as changes to the retinal and choroidal microstructure contributes to the recovery of visual function after surgery.

## 1. Introduction

The idiopathic macular hole (IMH) seriously affects central vision [1, 2]. Surgery to close the hole can improve the patient's vision [3]. Studies have reported that the rate of successful macular hole treatment is as high as 85–100% through vitrectomy [4]. Although the macular hole anatomical closure rate is very high, the corresponding improvements in visual acuity are not ideal. Improvements in visual acuity after macular hole surgery may be related to changes to the hole morphology and fundus retinal and choroidal microstructure [4–7]. Although the macular hole may achieve good anatomical closure after surgery, differences in postoperative visual function are observed. Evidence suggests that at least half of all macular hole patients still have low vision [8, 9]. Even individuals with similar preoperative vision exhibit significant differences in visual function after surgery, and it is difficult to explain the differences between visual function recovery and anatomical recovery.

In addition to the assessment of visual acuity, microperimetry (MP1) [10–12] can be used to analyse retinal sensitivity to assess visual function. OCT [2, 13–16] allows for analysis of the morphology of a macular hole and its derivative indices (maximum basal diameter, maximum basal diameter, and macular hole cystoid height). Furthermore, retinal thickness and inner or outer retinal capillary zones can be easily observed with OCTA [16–21]; these measures can be used to evaluate the changes in the deep or superficial retinal capillaries and the foveal avascular zone (FAZ) as well as the relationships between these changes and visual function after macular hole surgery. Reibaldi et al. [5] argued that the choroidal thickness of macular hole eyes is significantly lower than that of fellow eyes (i.e., the patient's healthy eye) and normal eyes, and thus, the recovery of vision might be related to changes in the choroidal thickness. Kim [6] argued that the choroidal thickness might be the protective factor for final BCVA and changes in vision after surgery, with patients with higher choroidal thickness in the macular hole eye achieving better postoperative vision.

In patients with the macular hole, visual function and the morphology of the retina and choroid are different from normal eyes [4–6]. Although visual function and morphology can approach that of normal eyes after surgery to correct the hole, the relationships between visual function and the retinal and choroid microstructure in patients with the idiopathic macular hole require further exploration. Through OCT, OCTA, and MP-1, this study comprehensively analysed the changes in visual function and the retinal and choroidal morphology to investigate the relationships between these factors.

## 2. Methods

### 2.1. Subjects

The sample for this study comprised 16 patients who were diagnosed with the idiopathic full-thickness macular hole in the Wenzhou Medical University Eye Hospital from June 2018 to July 2019. All patients with the macular hole underwent vitrectomy combined with internal limiting membrane peeling. All patients agreed to participate and provided written informed consent. The inclusion criteria were as follows: diagnosed with the idiopathic full-thickness macular hole (Gass classification [18]); spherical equivalent refraction −3.0O–+3.00D; no history of eye trauma or surgery; no history of hypertension, diabetes, or other serious systemic diseases; high-quality retinal and choroid images able to be obtained from the patient and the patient is able to cooperate with the examination. This study was performed in accordance with the Helsinki Declaration and was approved by the hospital's ethics committee (KYK[2018]52).

### 2.2. Instruments

The following instruments were used: Heidelberg OCT (Germany), AngioVue OCTA Optovue (USA), IOL-Master (Zeiss), MP-1 (NIDEK, Italy), comprehensive refractometer, and EDTRS visual chart. The Optical Laboratory (Wenzhou Medical Eye Hospital) provided special OCTA image analysis software.

### 2.3. Visual Function

Patients underwent comprehensive ophthalmologic examinations, including BCVA measured through EDTRS visual charts, a slit-lamp examination, indirect ophthalmoscopy, and intraocular pressure (IOP) measurement by using a noncontact tonometer (KT-500 Japan) at the preoperative and postoperative visits. Retinal sensitivity was measured by an MP-1 microperimetry examination [10–12]: the retinal sensitivity is determined within 10° of the gaze point, 0–4° is the sensitivity of the central retina, and 4–10° is the sensitivity of the peripheral retina.

### 2.4. Choroid

**Figure 1:**
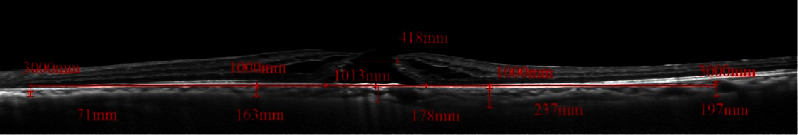
The minimum diameter, base diameter, and choroidal thickness (the choroidal thickness included nine positions: the subfoveal choroidal thickness and eight other superior, inferior, nasal, and temporal positions about 1 mm and 3 mm below the fovea) were measured.

**Figure 2:**
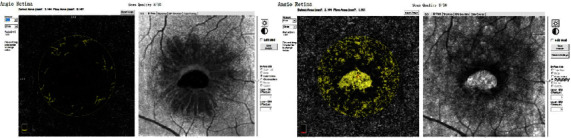
The blood flow areas of the outer retinal layer and choroid capillary layer.

OCT images were collected from 9 a.m. to 11 a.m.; clear and full-thickness choroidal images [2, 13–16] were obtained using a Heidelberg OCT (EDI mode). Then, the mean values of the minimum diameter and base diameter of the macular hole in the horizontal and vertical scanning directions were calculated. The thickness of the choroid was measured by two independent researchers, and the average value of the two measurements was used for analysis. The measurement positions were the subfoveal choroidal thickness (SFCT) and eight positions in the superior, inferior, nasal, and temporal directions, approximately 1 mm and 3 mm below the fovea (Figure 1). The minimum diameter and basal diameter in the horizontal and vertical direction were determined, and the mean value was calculated. The total cystic height within 1 mm diameter of the hole centre was measured by the Heidelberg OCT, and the macular hole cystoid height area index (MCHAI) (total cystoid height/basal diameter), macular hole index (MHI) (average height/basal diameter), diameter hole index (DHI) (minimum diameter/basal diameter), and tractional hole index (THI) (average height/minimum diameter) were calculated. The peripheral blood vessel density of the choroid capillary layer was measured by OCTA (Figure 2).

### 2.5. Retina

**Figure 3:**
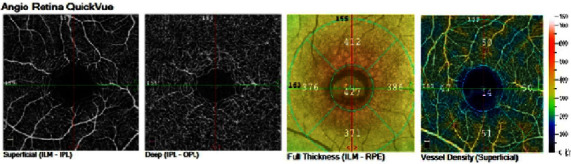
The full thickness of the retina and the blood flow density of the superficial retinal capillaries.

**Figure 4:**
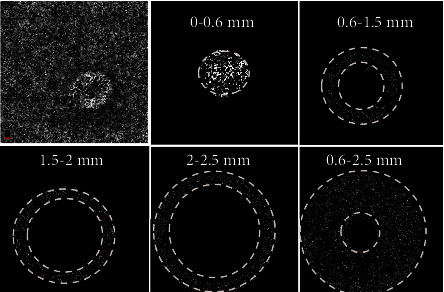
The retinal superficial or deep capillaries, the FAZ, and the choroidal blood vessel superficial capillary density (in the 0–0.6 mm, 0.6–1.5 mm, 1.5–2.0 mm, 2.0–2.5 mm, and 0.6–2.5 mm diameter areas).

The condition of the retina [16–21] was examined with an OCTA (Figures 3 and 2), and the blood flow density of the superficial retinal capillaries (ILM: internal limiting membrane to IPL: inner plexiform layer), the blood flow areas of the FAZ, and the full retinal thickness were measured by scanning a 3^∗^3 mm^2^ area of the macula. Then, the retinal superficial or deep capillaries (IPL: inner plexiform payer to OPL: outer plexiform layer), the FAZ, and the blood vessel density of the choroid capillary layer (within the 0–0.6 mm, 0.6–1.5 mm, 1.5–2.0 mm, 2.0–2.5 mm, and 0.6–2.5 mm diameter areas) were measured by MATLAB, and OCTA image analysis software developed in the OCT laboratory (Figure 4). Finally, the blood vessel density of the choroid capillary was calculated.

### 2.6. Statistical Analysis

The data are expressed as the mean ± standard deviation (SD). The differences between the three groups were analysed by ANOVA (an independent samples *T*-test was used for variance heterogeneity). Pearson correlation analysis was performed to examine the relationships between the variables.*P* < 0.05 was considered statistically significant. SPSS 25 software was used for all statistical analyses (SPSS, IBM, Chicago, IL, USA).

## 3. Results

### 3.1. Subjects

#### 3.1.1. Basic Parameters

The sample comprised 16 patients with the macular hole (5 males and 11 females, mean age of 64.25 ± 6.96 years, and range from 48 to 71 years) and 16 patients with normal eyes (4 males and 12 females, mean age of 63.25 ± 5.15 years, and range from 48 to 71 years). There were no statistically significant differences in age and sex between these two groups (*P* > 0.05). The basal diameter was 885 ± 255 *μ*m (172–1210 *μ*m), the minimum diameter was 529 ± 168 *μ*m (117–769 *μ*m), the DHI was 0.61 ± 0.13 (0.40–0.85), the average height of the retina within 1 mm of the hole was 419 ± 48 *μ*m (309–514 *μ*m), the total cystic height within 1 mm diameter of the hole centre was 610 ± 226 *μ*m (191–1028 *μ*m), and the MCHAI was 0.79 ± 0.54 (0.22–2.61).

#### 3.1.2. Visual Function

The BCVA of the macular hole eyes gradually improved after surgery; the improvement in these eyes was statistically significant, *P* < 0.05. The BCVA of fellow eyes (nonaffected eyes of the macular hole patients) exhibited no statistically significant difference compared to normal eyes, *P* > 0.05. The retinal sensitivity of the macular hole eyes (0°–4°) gradually increased after surgery; these changes were also statistically significant, *P* < 0.05. There were no significant differences between the macular hole eyes three months after surgery relative to the fellow eyes and the normal eyes, *P* > 0.05. There were no significant differences in peripheral retinal sensitivity (4°–10°) among the groups, *P* < 0.05. These findings are presented in Table 1.

#### 3.1.3. Retina

The preoperative retinal thickness of the hole eyes was statistically higher than the postoperative retinal thickness of the hole eyes, the retinal thickness of the fellow eyes, and the retinal thickness of the normal eyes, *P* < 0.05; however, there were no significant differences between the postoperative retinal thickness of the hole eyes, the retinal thickness of the fellow eyes, and the retinal thickness of the normal eyes, *P* > 0.05. The preoperative central retinal blood density of the superficial and deep capillaries was statistically lower than the postoperative central retinal blood density, *P* < 0.05. The FAZ of the hole eyes in both the superficial and deep layers was significantly lower than the fellow eyes and the normal eyes, *P* < 0.05. These results are presented in Table 2.

#### 3.1.4. Choroid

The preoperative superficial blood flow areas of the choroid capillary layer in the hole eyes were statistically lower than the postoperative superficial blood flow areas of the hole eyes, the superficial blood flow areas of the fellow eyes, and the superficial blood flow areas of the normal eyes, *P* < 0.05; however, there were no statistically significant differences in the postoperative superficial blood flow areas of the hole eyes, the superficial blood flow areas of the fellow eyes, and the superficial blood flow areas of the normal eyes, *P* > 0.05. The peripheral choroidal blood vessel superficial capillary density of the hole eyes was significantly lower than that of the fellow eyes and normal eyes, *P* < 0.05. The preoperative subfoveal choroidal thickness of the hole eyes was significantly lower than the postoperative subfoveal choroidal thickness of the hole eyes, the fellow eyes, and the normal eyes, *P* < 0.05; however, there were no statistically significant differences in the postoperative subfoveal choroidal thickness of the hole eyes, the fellow eyes, and the normal eyes, *P* > 0.05 ( Table 3).

#### 3.1.5. Correlation Analysis

**Figure 5:**
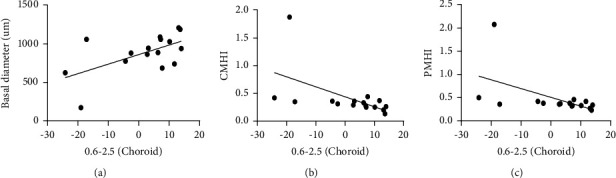
Correlation analysis between the change in the peripheral blood vessel density of the choroid capillary layer and the morphology of the macular hole, (a) correlation between the change in the peripheral blood vessel density of the choroid capillary layer and the basal diameter, (b) correlation analysis between the change in the peripheral blood vessel density of the choroid capillary layer and the CMHI, and (c) correlation analysis between the change in the peripheral blood vessel density of the choroid capillary layer and the PMHI).

The change in BCVA was significantly correlated with the basal diameter (*r* = 0.677 and *P* = 0.004), the minimum diameter (*r* = 0.585 and *P* = 0.017), the MCHAI (*r* = −0.618 and *P* = 0.011), the central MHI (*r* = −0.727 and *P* = 0.001), the peripheral MHI (*r* = −0.758 and *P* = 0.001), the central THI (*r* = −0.717 and *P* = 0.002), the peripheral THI (*r* = −0.725 and *P* = 0.001), and the change in the peripheral blood vessel density of the choroid capillary layer (*r* = 0.585 and *P* = 0.0017). The change in the central retinal sensitivity was correlated with the change in the superficial FAZ (*r* = 0.520 and *P* = 0.039), the change in the retinal superficial peripheral blood flow density (*r* = −0.503, *P* = 0.047), the change in the deep FAZ (*r* = 0.599 and *P* = 0.014), and the change in the retinal deep peripheral blood flow density (*r* = −0.601 and *P* = 0.014), as shown in Table 4. The change in the peripheral blood vessel density of the choroid capillary layer was correlated with the change in the basal diameter (*r* = 0.592 and *P* = 0.016), the CMHI (*r* = −0.548 and *P* = 0.028), and the PMHI (*r* = −0.531 and *v*= 0.034), as shown in Figure 5.

## 4. Discussion

This study found that BCVA and central retinal sensitivity of the macular hole gradually improved after vitrectomy combined with internal limiting membrane stripping and tamponade. Chen et al. [10–12] also confirmed that the visual function of the macular hole eye was improved after surgery. The results of the current study also indicated that the thickness of the retina in the central and peripheral zones gradually decreased with closure of the hole, and the cyst within 1 mm diameter of the hole centre disappeared after the hole closed. The decrease in the retinal thickness is consistent with the research of Imamura [22], with a decrease in the retinal thickness and the disappearance of the cyst appearing to contribute to the improvement in visual function. Moreover, the superficial blood flow areas of the choroid capillary layer, the choroidal blood vessel superficial density capillary, and the choroidal thickness exhibited significant improvements after macular hole surgery. The studies of Reibaldi et al. [5] and Kim et al. [6] also found that the choroidal thickness of the hole eyes was lower than that of the fellow eyes and normal eyes. The improvements in visual function and fundus microstructure in the retina and choroid of the hole eyes after macular hole surgery resulted in function and microstructure characteristics that were close to those of the fellow eyes and normal eyes.

The correlation analysis indicated that the postoperative change in BCVA was correlated with the morphology of the macular hole and its derivative indices. The ∆BCVA was negative as it is calculated as the three-month BCVA minus the preoperative BCVA. The smaller basal diameter, smaller minimum diameter, larger MCHAI, larger central or peripheral MHI, larger central or peripheral THI, and smaller peripheral blood vessel density of the choroid capillary layer all contributed to the improvement in BCVA. Macular holes seriously damage vision, and patients with macular holes with a smaller basal diameter and smaller minimum diameter may exhibit greater improvements in visual function following surgery. This is consistent with the research of Venkatesh et al. [2]. The presence of retinal cysts is indicative of greater anteroposterior tractional forces and taller macular holes. The anteroposterior traction forces could be represented by the macular hole height and nasal and temporal arm lengths, and the diameter hole index evaluates the distance between the edges of the hole. The MHI, THI, and MCHAI could help analyse the anteroposterior tractional forces caused by cysts, and the lower index might obtain better restoration of visual function.

Thickening of the retina in the macular hole may be attributed to the anteroposterior and tangential traction caused by incomplete vitreoretinal detachment, the presence of the vitreous cortex and epimacular glial tissues, or retinal swelling with subretinal fluid components [1–4]. The MHI includes both the horizontal and vertical dimensions of the macular hole, representing the putative tangential and anteroposterior vitreomacular traction or retinal hydration; a higher MHI value indicates a smaller basal diameter and a larger height [2]; a higher MCHAI and THI also represent smaller horizontal dimensions and a larger height. The effusion separates the retinal neurosensory layer from the retinal pigment epithelium, and the oxygen transmission from the choroidal capillaries to the deep retina is blocked by the effusion, resulting in hypoxia of the retinal neurosensory layer. After surgery, the effusion disappears and the hypoxia of the retina is ameliorated, thus improving visual function. Thus, a patient with a macular hole with a smaller basal diameter and greater height might achieve better improvement in visual function after surgery.

The retinal thickness was decreased in the central region, upper and lower nasal regions, and temporal region after surgery. Thinning of the retina in the macular hole mainly occurs in the nerve fibre layer and ganglion cell layer. Ozdemir et al. [23] found that the nerve fibre layer was significantly thinner in the temporal, upper, and lower sides, which might be caused by mechanical damage due to internal limiting membrane stripping. After surgery, the retinal cells are affected by local inflammation, microcirculation ischaemia and stretching, or the toxicity of macular stain; this can lead to damage to the nerve fibre layer and ganglion cell layer, resulting in a reduction in retinal thickness.

The results of this study indicated that the change in central retinal sensitivity was correlated with changes in the superficial or deep FAZ and the retinal superficial or deep peripheral blood flow density. After the macular hole is closed, the FAZ reduces. The main reason for the decrease in the FAZ is the surgical stripping of the inner limiting membrane, which improves the closure rate of the hole. After surgery, the retinal cells migrate to fill the gap caused by the macular hole, the tissue increases in the macular hole, and the FAZ decreases [7, 24–26]. Thus, it can be inferred that a greater reduction in the FAZ relates to better recovery of the central retinal tissue and a greater improvement in visual function.

The central superficial retinal blood flow density was significantly increased, and the peripheral superficial retinal blood flow density was decreased one month after surgery. This suggests that the month after surgery is the critical period for closure of the macular hole. The morphology and function of the central retina recovered gradually; the peripheral retinal blood flow slowly migrated to the central part of the retina, contributing to the morphological and functional recovery of the macular hole. Thus, the peripheral retinal blood flow decreased, and the central blood flow increased. The decrease in the FAZ also reflects this phenomenon. Although the retinal blood flow increased from one month to three months after surgery, this difference was not statistically significant, presumably, because the hole was basically closed one month after surgery and the morphology tended to be stable. The retinal blood flow also tended to be stable; thus, there was no need for peripheral blood flow support.

This study revealed that the subfoveal, temporal, and superior choroidal thickness were greatest, followed by the inferior choroidal thickness; the nasal choroidal thickness was the thinnest. This is consistent with previous studies [5–8]. It is speculated that the region where the nasal optic papilla is located has fewer choroidal vessels than the other regions. Ozdemir et al. [23] showed that the choroidal thickness became thinner after surgery. However, some studies have found only significant subfoveal choroidal thickness thinning, while other parts of the choroidal thickness exhibit little statistical change after surgery [17, 27]. Although the relationship between thinning of the choroid and the change in visual function is unclear, most studies supported thickening of the choroid after surgery [7, 13, 17, 24, 26–29].

Zhang et al. [13] reported that the subfoveal choroidal thickness is lower in macular hole eyes than normal eyes, that the change in the choroidal blood flow occurs before complete formation of the macular hole, and that thinning of the choroidal thickness might be caused by the macular hole. Aras et al. [30] and Morgan and Schatz [31] suggested that choroidal hypoperfusion is the result of pore formation rather than the cause of pore formation. Zeng et al. [32] reported that thinning of the choroidal thickness in the macular hole is not limited to the fovea but occurs in all 6-mm-diameter regions of the posterior pole. This suggests that changes in the choroidal blood flow might be one reason for the occurrence of macular holes. This study found no relationship between thinning of the choroidal thickness and changes in visual function; however, ∆BCVA was correlated with a change in the peripheral blood vessel density of the choroid capillary layer, and the morphology of the macular hole was correlated with changes in the peripheral blood vessel density of the choroid capillary layer but not with thinning of the choroidal thickness. It is speculated that the peripheral blood flow density of the choroid capillary layer may be more sensitive to changes in the choroidal thickness supporting the retinal vessels. A greater decrease in the peripheral blood vessel density may support more blood flow to the retina, thus contributing to better recovery of visual function.

There are several limitations of this study that should be noted. First, the number of cases was limited. The study recruited more than 40 individuals with the idiopathic macular hole, but most of them were excluded due to the fit and poor image quality from the optical examinations. Second, the central retina and choroid could not be examined directly due to the macular hole; thus, the peripheral changes in the retina and choroid were examined. Third, because of the high loss of participants, this study could only analyse changes three months after surgery rather than the trend in visual recovery. BCVA can also represent recovery of visual function to a certain extent.

## 5. Conclusion

The recovery of visual function is affected by many factions after macular hole surgery. The morphology of the macular hole as well as changes in the retinal and choroidal microstructure all contribute to the recovery of visual function after surgery. The MHI, THI, and MCHAI could help analyse the anteroposterior tractional forces caused by cysts, and the lower index might obtain better restoration of visual function. Macular holes with a smaller basal diameter and greater height might obtain more blood flow support from the choroid, especially from the peripheral blood vessel of the choroid, allowing better recovery and greater improvements in visual function.

## Figures and Tables

**Table 1:** Differences in BCVA and retinal sensitivity among the eyes.

	Macular hole eyes			Fellow eyes	Normal eyes	*P* value
	Pre-surgery	One month	Three months			
BCVA	1.02 ± 0.22	0.66 ± 0.27	0.47 ± 0.27	0.13 ± 0.10	0.04 ± 0.05	<0.001
Retinal sensitivity (dB)						
0°–4°	8.76 ± 3.27	12.22 ± 3.30	14.88 ± 2.87	16.89 ± 2.15	16.15 ± 3.25	<0.001
4°–10°	15.12 ± 3.49	15.25 ± 2.41	16.41 ± 3.15	17.66 ± 1.45	16.82 ± 2.77	0.109
0°–10°	13.29 ± 3.12	14.37 ± 2.57	15.97 ± 2.82	17.44 ± 1.62	16.63 ± 2.89	0.001

*Note.* BCVA: best-corrected visual acuity.

**Table 2:** Differences in the retinal thickness and blood density among the eyes.

Retina	Macular hole eyes			Fellow eyes	Normal eyes	*P* value
	Pre-surgery	One month	Three months			
Retinal thickness (*μ*m)						
Centre	393 ± 53	286 ± 38	279 ± 48	245 ± 22	236 ± 22	<0.001
Paracentral	365 ± 31	339 ± 20	331 ± 24	317 ± 24	315 ± 22	<0.001
Superior	363 ± 34	339 ± 24	331 ± 27	321 ± 28	322 ± 24	<0.001
Inferior	364 ± 30	338 ± 19	332 ± 20	319 ± 17	303 ± 74	0.001
Nasal side	379 ± 36	355 ± 23	349 ± 29	308 ± 39	321 ± 19	<0.001
Temporal side	354 ± 25	320 ± 18	309 ± 24	314 ± 18	312 ± 17	<0.001
Blood density of the superficial retinal capillaries (%)						
0–0.6 mm	6.56 ± 10.97	26.67 ± 14.35	13.57 ± 14.59	4.66 ± 8.90	0.02 ± 0.03	<0.001
0.6–1.5 mm	52.44 ± 3.25	54.12 ± 1.331	53.12 ± 1.90	50.31 ± 1.90	48.31 ± 1.33	<0.001
1.5–2.0 mm	55.68 ± 0.95	54.92 ± 1.50	54.82 ± 1.14	55.38 ± 1.80	54.58 ± 1.18	0.315
2.0–2.5 mm	55.22 ± 0.75	55.01 ± 1.32	54.54 ± 1.39	54.43 ± 1.25	53.76 ± 1.48	0.193
0.6–2.5 mm	54.43 ± 1.32	54.66 ± 1.33	54.16 ± 1.13	53.39 ± 1.40	52.25 ± 1.13	0.014
Superficial FAZ	0.32 ± 0.12	0.16 ± 0.10	0.25 ± 0.11	0.40 ± 0.14	0.47 ± 0.04	<0.001
Blood density of the deep retinal capillaries (%)						
0–0.6 mm	6.32 ± 10.28	26.07 ± 14.42	13.44 ± 14.55	4.73 ± 9.48	0.03 ± 0.05	<0.001
0.6–1.5 mm	53.79 ± 3.58	55.83 ± 2.37	55.28 ± 2.65	52.72 ± 3.73	50.84 ± 2.69	0.019
1.5–2.0 mm	56.24 ± 1.11	56.00 ± 2.42	56.64 ± 1.40	56.58 ± 2.37	55.05 ± 2.23	0.649
2.0–2.5 mm	55.35 ± 1.13	55.39 ± 2.24	55.74 ± 1.47	55.24 ± 2.09	54.26 ± 1.46	0.742
0.6–2.5 mm	55.11 ± 1.66	55.72 ± 2.29	55.86 ± 1.59	54.83 ± 2.12	53.40 ± 2.06	0.226
Deep FAZ	0.28 ± 0.17	0.10 ± 0.08	0.22 ± 0.09	0.34 ± 0.14	0.37 ± 0.10	<0.001

**Table 3:** Differences in the choroidal thickness and blood density among the eyes.

Choroid	Macular hole eyes			Fellow eyes	Normal eyes	*P* value
	Pre-surgery	One month	Three months			
Flow area (mm^2^)	1.31 ± 0.30	1.76 ± 0.15	1.87 ± 0.12	1.95 ± 1.33	1.93 ± 1.22	<0.001
Blood vessel superficial capillary density (%)						
0–0.6 mm	73.72 ± 13.50	60.59 ± 8.59	58.84 ± 4.08	61.10 ± 7.73	63.61 ± 5.31	<0.001
0.6–1.5 mm	39.48 ± 12.99	48.46 ± 7.13	49.54 ± 5.68	56.02 ± 8.00	54.62 ± 2.77	<0.001
1.5–2.0 mm	49.42 ± 12.06	49.57 ± 7.85	51.50 ± 4.79	56.27 ± 6.65	54.49 ± 2.53	0.014
2.0–2.5 mm	59.52 ± 10.97	52.39 ± 6.80	54.33 ± 3.91	58.89 ± 5.73	56.24 ± 2.38	0.044
0.6–2.5 mm	49.82 ± 11.70	50.20 ± 7.13	51.69 ± 4.40	57.19 ± 6.63	55.20 ± 2.12	0.028
Choroidal thickness (um)						
SFCT	190 ± 24	216 ± 28	221 ± 28	225 ± 29	229 ± 41	0.006
Superior 1 mm	193 ± 35	216 ± 30	218 ± 33	199 ± 44	234 ± 51	0.037
Inferior 1 mm	181 ± 38	199 ± 32	202 ± 39	192 ± 32	213 ± 52	0.207
Nasal 1 mm	172 ± 31	191 ± 38	187 ± 41	190 ± 63	208 ± 68	0.391
Temporal 1 mm	205 ± 29	227 ± 30	227 ± 38	225 ± 38	226 ± 40	0.329
Superior 3 mm	199 ± 43	210 ± 41	212 ± 48	207 ± 46	227 ± 64	0.603
Inferior 3 mm	170 ± 46	185 ± 45	185 ± 46	163 ± 47	206 ± 52	0.143
Nasal 3 mm	98 ± 33	106 ± 25	107 ± 32	103 ± 57	147 ± 52	0.008
Temporal 3 mm	193 ± 26	206 ± 30	215 ± 34	211 ± 32	211 ± 59	0.544

*Note.* Flow area: the blood flow areas of the choroid capillary layer; blood vessel density: the blood vessel density of the choroid capillary layer (diameter of 0–0.6 mm, 0.6–1.5 mm, 1.5–2.0 mm, 2.0–2.5 mm, and 0.6–2.5 mm); SFCT: subfoveal choroidal thickness.

**Table 4:** Correlation analysis between visual function and macular hole structure.

	∆BCVA		∆Retinal sensitivity (dB)				BCVA		Retinal sensitivity (dB)			
	*r*	*P*	0°∼4°		4°∼10°		*r*	*P*	0°∼4°		4°∼10°	
			*r*	*P*	*r*	*P*			*r*	*P*	*R*	*P*
Basal diameter (*μ*m)	0.677	0.004	0.225	0.401	−0.146	0.590	0.576	0.020	−0.634	0.008	−0.519	0.039
Minimum diameter (*μ*m)	0.585	0.017	0.014	0.960	−0.242	0.367	0.733	0.001	−0.567	0.022	−0.474	0.063
DHI	−0.081	0.766	−0.405	0.119	−0.209	0.438	0.304	0.252	0.076	0.780	0.018	0.948
Total cystic height (*μ*m)	0.256	0.339	0.154	0.568	0.156	0.565	0.113	0.677	−0.522	0.038	−0.336	0.204
MCHAI	−0.618	0.011	−0.245	0.359	0.043	0.874	−0.487	0.055	0.291	0.275	0.196	0.467
Central MHI	−0.727	0.001	−0.335	0.205	−0.296	0.266	−0.525	0.037	0.499	0.049	0.319	0.228
Central THI	−0.717	0.002	−0.181	0.502	−0.240	0.371	−0.653	0.006	0.558	0.025	0.385	0.141
Peripheral MHI	−0.758	0.001	0.021	0.938	0.021	0.938	−0.565	0.023	0.540	0.031	0.352	0.182
Peripheral THI	−0.725	0.001	0.086	0.751	0.080	0.769	−0.610	0.012	0.521	0.039	0.352	0.181
SFAZ	0.303	0.254	0.520	0.039	−0.181	0.501	−0.002	0.993	−0.050	0.856	−0.263	0.325
Retinal superficial peripheral blood flow density (0.6–2.5)	−0.288	0.279	−0.503	0.047	−0.105	0.700	0.067	0.806	0.113	0.677	0.164	0.545
DFAZ	0.453	0.078	0.599	0.014	−0.100	0.711	0.117	0.666	0.006	0.984	−0.134	0.621
Retinal deep peripheral blood flow density (0.6–2.5)	−0.311	0.241	−0.601	0.014	0.311	0.241	−0.026	0.925	−0.108	0.690	0.152	0.575
0.6–2.5 (choroid)	0.585	0.017	0.164	0.544	0.055	0.841	0.434	0.093	−0.635	0.008	0.473	0.064
SFCT	0.400	0.125	−0.142	0.601	0.017	0.950	0.313	0.238	−0.111	0.683	0.041	0.880

*Note.* DHI: minimum diameter and basal diameter ratio; MCHAI: total cystic height and basal diameter ratio; central MHI: central retinal thickness and basal diameter ratio; central THI: central retinal thickness and minimum diameter ratio; peripheral MHI: paracentral retinal thickness and basal diameter ratio; peripheral THI: paracentral retinal thickness and minimum diameter ratio; ∆: change between presurgery and three months postsurgery; SFAZ: superficial retinal capillary fovea avascular zone; DFAZ: deep retinal capillary fovea avascular zone; SFCT: subfoveal choroidal thickness.

## Data Availability

The data used to support the findings of this study are available from the corresponding author (Dan Xu) upon request.
